# Factors influencing risky sexual behaviour among Mozambican miners: a socio-epidemiological contribution for HIV prevention framework in Mozambique

**DOI:** 10.1186/s12939-017-0674-z

**Published:** 2017-10-10

**Authors:** Emilia Martins-Fonteyn, Osvaldo Loquiha, Cynthia Baltazar, Subash Thapa, Makini Boothe, Ines Raimundo, Niel Hens, Marc Aerts, Herman Meulemans, Olivier Degomme, Edwin Wouters

**Affiliations:** 1 Research Centre for Longitudinal and Life Course Studies and Department of Sociology, University of Antwerp, City Campus, Prinsstraat 13, BE-2000 Antwerp, Belgium; 2 Interuniversity Institute for Biostatistics and statistical Bioinformatics, University of Hasselt, Hasselt, Belgium; 3 Department of Mathematics and Informatics, University Eduardo Mondlane, Maputo, Mozambique; 4 Surveillance Department at National Institute of Health, Ministry of Health, Maputo, Mozambique; 5 Department of Public Health, KU Leuven, Leuven, Belgium; 6 Department of Public Health, Institute of Tropical Medicine, Antwerp, Belgium; 7 University of California, San Francisco – USA, of Global Health Sciences, Maputo, Mozambique; 8 Faculty of Social Sciences, University Eduardo Mondlane, Maputo, Mozambique; 9 Centre for Health Economic Research and Modelling Infectious Diseases, Vaccine and Infectious Disease Institute, University of Antwerp, Antwerp, Belgium; 10 International Centre for Reproductive Health (ICRH), University of Gent, Gent, Belgium

**Keywords:** HIV infection, Risky sexual behaviour, Mozambican miners

## Abstract

**Background:**

Information dealing with social and behavioural risk factors as well as their mechanisms among Mozambican migrants working in South African mines remains undocumented. This study aims to understand the various factors influencing HIV-related risk behaviours and the resulting HIV positive status of Mozambican miners employed by South African mines. This analysis was undertaken in order to inform a broader and more effective HIV preventive framework in Mozambique.

**Method:**

This study relied upon data sourced from the first Integrated Biological and Behavioural Survey among Mozambican miners earning their living in South African mines. It employs quantitative techniques using standard statistical tools to substantiate the laid-down objectives. The primary technique applied in this paper is the multivariable statistical method used in the formulation and application of a proximate determinants framework.

**Results:**

The odds of reporting one sexual partner were roughly three times higher for miners working as perforators as opposed to other types of occupation. As well, the odds of condom use – always or sometimes – for miners in the 31-40 age group were three times higher than the odds of condom use in the 51+ age group. Miners with lower education levels were less likely to use condoms.

The odds of being HIV positive when the miner reports use of alcohol or drugs (sometimes/always) is 0.32 times lower than the odds for those reporting never use of alcohol or drugs. And finally, the odds of HIV positive status for those using condoms were 2.16 times that of miners who never used condoms, controlling for biological and other proximate determinants.

**Conclusion:**

In Mozambique, behavioural theory emphasising personal behavioural changes is the main strategy to combat HIV among miners. Our findings suggest there is a need to change thinking processes about how to influence safer sexual behaviour. This is viewed to be the result of a person’s individual decision, due to of the complexity of social and contextual factors that may also influence sexual behaviours. This only stresses the need for HIV prevention strategies to exclusively transcend individual factors while considering the broader social and contextual phenomena influencing HIV risk among Mozambican miners.

## Background

Sub-Saharan Africa is the region hardest hit by the HIV epidemic. Of the 35 million people living with HIV worldwide, 24.7 million are living in Sub-Saharan Africa – nearly 71% of the global total. Mozambique is one of the ten countries accounting for 81% of all people living with HIV in the region [1].

It has an HIV prevalence of 11.5% in adult population (15-49 years old), and 320 Mozambicans are infected by HIV every day. The burden of the epidemic varies considerably across the various regions of the country. In the south, where the level of migrant work is very high, HIV is spreading the fastest. With a prevalence of 17.8%, the south has the highest HIV rate in the country. Within the south, Gaza province has the highest prevalence at 25%. This is more than twice the national prevalence rate. Shockingly, this is almost 5 times higher than that of the north of Mozambique where the prevalence rate is just 5.6%. In the central region, HIV prevalence is 12.5% due to the transport corridors linking Beira Port with neighbouring countries [2].

In order to address the HIV epidemic, the country adopted the National Strategic Plan, which incorporates four generations (National Strategic Plan I – 2000-2002, National Strategic Plan II – 2005-2009, National Strategic Plan III – 2010-2014, and National Strategic Plan IV – 2015-2019). The country also developed other instruments, such as the Strategic Plan for the Health Sector, 2004. This portion of the strategy stresses the use of a contextually relevant approach to communication as well as creating guidelines for national response [3].

Prevention efforts have been dominated by interventions aimed almost exclusively at the individual level. Such interventions are based on theories of individual psychology, primarily linking HIV transmission to behavioural and cognitive factors such as knowledge, attitudes, beliefs, skills, etc. [4]. This approach has been helpful in establishing baseline risk data for various groups as well as identifying some reasonably predictive risk factors. However, it has also revealed just how limited the available knowledge is with regard to cultural, social-relational, and sexual nature of the risk of contracting HIV. As well as demonstrating the limitations in conducting cross-cultural research, it has revealed diminishing returns with respect to infection rates and levels of safer sex [4].

In Mozambique, despite these efforts, HIV/AIDS continues to have a devastating effect on all aspects of social and economic life. Although, the overall number of new infections is decreasing in the country, in the northern region, where the actual prevalence is the lowest, the incidence rates are increasing. In the south and central parts of the country, HIV prevalence remains very high [3].

The HIV is a society-wide health problem, however the National Strategic Plan recognizes that certain population groups, such as Mozambican miners working in South Africa are especially vulnerable. This is due to their high risk of HIV infection, as a result of socioeconomic, cultural, or behavioural factors – therefore requiring special attention [3].

The majority of Mozambicans from the south of the country – documented and undocumented – work on commercial farms, mines, and construction sites in South Africa [5]. Therefore, in Mozambique, the epidemiology of HIV/AIDS seems closely linked to migration. The largely seasonal or temporary character of migration to South Africa means migrants return home to their families on a regular basis. This has facilitated the rapid spread of the virus [6].

Even in the current phase of the epidemic, with high prevalence levels on both sides of the border – South Africa displays similar prevalence levels (18%) as the South of Mozambique [7]. This evidence suggests migrants are still more vulnerable to HIV-infection than their non-migrant counterparts, underscoring the need for research on the determinants of this vulnerability [8].

In Mozambique, the first Integrated Biological and Behavioural Survey (IBBS) among Mozambican miners working in South African mines, was conducted in 2012. It revealed miners from the south of Mozambique had an estimated HIV prevalence of 22% [9]. The IBBS brings new information regarding biological and behavioural factors affecting miners’ HIV risks. However, information on how social and contextual determinants affect the risk of acquiring HIV by shaping patterns of miners’ susceptibility and vulnerability remains undocumented.

Therefore, this study aims to uncover and disentangle the various elements influencing HIV-related risk behaviours as well as the resulting HIV positive status of Mozambican miners working in South African mines. Our objective is to better inform a broader and more effective HIV preventive framework. The complex understanding of the ways the migratory system influences risky sexual behaviour of the miners requires a multilevel approach with the potential to deal with multiple factors ranging from individual, contextual to behavioural determinants [10].

In this context, the use of a proximate determinants framework offers a better opportunity to study different level factors, social, contextual and individual, and enrich our understanding of the relative strengths of various factors shaping the HIV epidemic. At same time, it provides explanations of how wide-ranging factors affect vulnerability to HIV in a specific population [10].

In this context, a proximate determinants framework is a valuable method to analyse factors affecting HIV infection, and the most appropriate to answer the following questions:

1. What are the factors influencing risky sexual behaviour among Mozambican miners?
2. How does migration affect these risks?

## Methods

This study uses data from the first IBBS among Mozambican miners. It employs quantitative techniques using standard statistical tools to substantiate the laid-down objectives. The major technique employed in this paper is a multivariable statistical method used in the formulation and application of the proximate-determinants framework.

### Framework

Because of the limited research on sociodemographic and other underlying factors associated with HIV transmission among Mozambican miners, we utilised the proximate-determinants framework developed by Boema and Weir to assess the various factors influencing HIV-related risk behaviours among mineworkers [11]. Originally, Davis and Blake developed proximate-determinants as an analytical framework for the comparative study of the sociology of fertility [11].

**Fig. 1:**
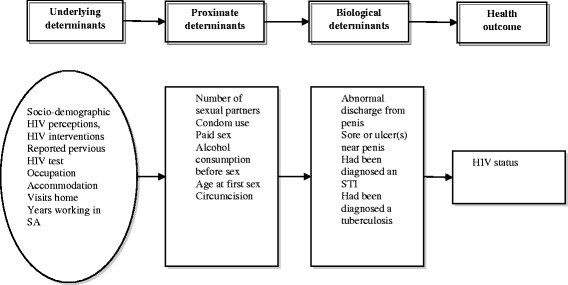
Proximate-Determinants Framework. Source: Adapted by the original, Sharma et al. 2013:550

For the purpose of this analysis, we assumed the underlying social, economic, and environmental determinants – independent variables – must operate through proximate determinants factors in order to affect biological dynamics and health outcomes with HIV status being the dependent variable. Figure 1 outlines the Proximate-Determinants Framework adapted to this analysis.

We considered underlying determinants as independent variables: socio-demographic, HIV interventions, reported previous HIV testing, risk perception and participation in HIV educational sessions. Socio-demographic factors are: age, education level, province of residence, marital status, as well as the number of wives in both Mozambique and South Africa.

For married miners or those who have steady relationships, we also looked into the country of residence of their partners. Moreover, underlying mining and mobility determinants were included occupation at the mine; accommodation during work at the mine; frequency of return visits to the home country and the number of years working in the mines.

Proximate determinants or behavioural factors are indicators of risky sexual behaviour used in this model. These include: the number of sexual partners; paid sex, condom use during the latest 3 sex acts; alcohol use before sex; age at the time of their first sexual encounter; and circumcision.

i) Multiple sexual partners and paid sex are included because having a greater number of sexual partners is associated with a risk of sexually transmitted infections – Sexually Transmitted Infections (STIs) including HIV [12].

ii) Frequency of condom use is a factor because consistent condom use is the main strategy aimed at preventing individuals from acquiring HIV through sexual intercourse [13].

iii) Alcohol and/or drug use before sex has been shown to influence the incidence of HIV infection and other STIs [14]. Male circumcision reduces the risk of HIV infection [15] and in Mozambique geography is highly associated with circumcision. Generally, rates of HIV infection in men are typically lower in the northern region, where the rate of male circumcision is much higher compared to the southern region [16].

Biological influences are co-factors for HIV. For instance, the risk of developing tuberculosis (TB) is estimated to be between 26 and 31 times greater in people living with HIV than among those without HIV infection [17]. STIs can increase susceptibility to HIV infection in several ways. For example, ulcerations compromise the skin’s physical barrier to infection and provide infectious agent easier access to the bloodstream [18]. Therefore, in this study, we used variables such as: reported STIs – abnormal discharge from the penis, sores or ulcers near the penis or the participant had been diagnosed as having an STI while as well as having been diagnosed with TB.

The result of the HIV test taken during the survey was classified as an outcome or dependent variable. Self-reported HIV tests – those taken previously by miners within the 12 months preceding the survey, were used as an underlying factor.

### Statistical analysis

This study investigates associations between the determinants using a multiple logistic regression. *P*-values at < 0.05 were considered statistically significant. Multiple logistic regression was applied to allow for adjustments in the association. This was done by using an odds ratio between the underlying and proximate determinants, as well as making use of the latter determinants as dependent variables. Logistic regressions were also applied to investigate the determinants of HIV status in building a 3 step model.

In the first step, (Model A), only biological effects – co-factors of HIV infection – were presented. These were then extended to include the proximate determinant in Model B and underlying determinants in Model C. Odds ratio and 95% confidence intervals for the three models were provided for inference. All analyses utilised the sample weights as provided by the survey design, and excluded missing values on the outcomes and independent variables with the assumption that absent values were completely at random. Of the 430 miners in the sample, approximately 26% were missing in the main outcome of HIV status, while 30.9% were missing either in the main outcome or the determinants variables.

### IBBS

The main objectives of the IBBS were to estimate the prevalence of HIV and associated risk behaviours as well as determine prevention indicators among Mozambican men who work in South African mines. This was done to assess the use of and access to health and social welfare programs among Mozambican workers in South African mines. This information was necessary to determine how to increase their coverage and uptake in Mozambique and in South Africa [9]. Specific information about recruitment criteria has been previously published.

### Methodology

The IBBS was a quantitative cross-sectional behavioural study, involving Mozambican miners working in South Africa who were recruited for interviews and HIV testing through Time-Location Sampling (TLS). This study was conducted between February and May 2012 by the Health National Institute (INS), in Ressano Garcia, Mozambique. The questionnaire has been previously published on the IBBS final report [9].

### Inclusion criteria

Miners recruited at TEBA Ltd. represented numerous mining operations, including gold, diamonds, coal, etc. To be eligible, a potential participant had to be a Mozambican man, 18 years of age or older, have signed a contract the same day as interviews to work at a South African mine, and have more than 12 months experience working in a South African mine.

### Sample size

Sample size estimates were based on the surveillance objective of detecting major changes in the epidemic over time – for example, between successive IBBS rounds. The key indicator selected was condom use at the time of last intercourse. This was determined to be 63% in a survey carried out in 2008 with members of communities adjacent to five South African mines.

The survey team sought to detect a 10% increase in the indicator of interest – from 63% to 73% usage – between consecutive surveys. Statistical significance was set at 0.05 and power was set at 0.80. The minimum sample size was estimated to be 340 and rounded to 400 for increased precision [9].

In addition, to ensure meeting the primary objective of estimating a prevalence of HIV with an acceptable confidence interval, a sample size of 398 miners was considered sufficient, around an estimated HIV prevalence of 25% on a population size of 40,000 [19]. A prevalence estimate of 25% was based on a 2003 cross sectional study of migrant and non-migrant miners working in gold mines in South Africa, in which 25.9% of migrant men were HIV positive [20]. The population size was based on figures from the Ministry of Labour which list the number of Mozambican mine workers in South Africa at 41,100 in 2009.

### Sample selection

The study site selection was based on the information that formative assessment revealed most Mozambican miners are affiliated with The Employment Bureau of Africa Ltd. (TEBA Ltd). Therefore, they must visit the TEBA Ltd. facilities in Ressano Garcia, which is a town on the border between Mozambique and South Africa. Here, the workers must sign/renew their contracts annually.

All Mozambican migrant miners are required to sign 12 month contracts. Approximately 150 mineworkers have their contracts signed and stamped daily. As well, each person must also receive a stamp at the local Mozambican Ministry of Labour (MITRAB), located near TEBA Ltd.

Based upon this information, the decision was made to recruit participants using TLS at MITRAB facilities in Ressano Garcia (i.e., a single venue) immediately following having their contracts stamped.

Sampling events consisted of 3 h blocks during the day and hours when the TEBA Ltd. and MITRAB offices in Ressano Garcia were open. Fifty sampling events were randomly selected from Monday to Friday to attain the target survey sample size of 400 miners. During sampling events, all potential participants were enumerated. As interviewers became available, potential participants were intercepted consecutively and invited to participate in the survey, until the sampling event ended. Informed consent was sought from all who were intercepted and found eligible.

### Biological test

HIV rapid testing was conducted on-site, following informed consent and pre-test counselling by certified counsellors, using two rapid tests. Nonreactive results in the first test were considered negative and reactive results were confirmed. Participants with reactive results on both tests were classified as HIV positive and participants with negative results were classified as indeterminate [9].

### Database acquisition

This secondary analysis was approved by IBBS Scientific Committee, who provided the dataset for analysis.

## Results

The present study consisted of 432 participating Mozambican miners. The majority of these Mozambican miners were 40-50 years old, married or in a union, and 76% of them have one wife, who lives in Mozambique. They mainly, 42%, originated from Gaza Province, in the south of Mozambique and 79% of them have primary level of education. The majority, 53%, work as engine drivers in South African mines, and 66% have worked as labourers/miners from 10 to 24 years (Tables 1 and 2).

**Table 1:** Underlying determinants^a^ (socio-demographic and economic characteristics) among miners

		Number (430)	Percentage
*Socio-demographic characteristics*			
Age group (years)	18 – 30	28	6.5
	31 – 40	144	33.5
	41 – 50	131	30.5
	≥ 51	127	29.5
Marital status	Never married	3	0.7
	Married/Union	414	96.3
	Divorced/Widow	13	3.0
Number of wives in South Africa (SA) and Mozambique (Moz)	No wife	16	3.7
	One in SA	4	0.9
	One in Moz	328	76.3
	More than 1 in Moz	48	11.2
	One in SA and Moz	34	7.9
Province of residence in Mozambique	Maputo	83	19.3
	Gaza	182	42.3
	Inhambane	154	35.8
	Outro	11	2.6
*Socio-economic characteristics*			
Education level	No schooling	25	5.8
	Primary	341	79.3
	Secondary	64	14.9
Occupation	Engine driver	229	53.3
	Perforator	88	20.5
	Supervisor	78	18.1
	Other	35	8.1
*Attitudes/ intervention programs on HIV*			
Tested for HIV previously (12 months before survey)	No	48	11.2
	Yes	382	88.8
Result of last HIV test (12 months before survey)	Negative	345	92.7
	Positive	27	7.3
Participated in an HIV lecture	No	88	20.5
	Yes	342	79.5
Perceived risk of contracting HIV	None	51	12.3
	Low	93	22.4
	Moderate	139	33.5
	High	105	25.3
	HIV+	27	6.5
*Mining and mobility*			
Accommodation in SA	Hostel	177	41.2
	Family	84	19.5
	Girlfriend/friend/colleague	43	10.0
	Alone	126	29.3
Years working at the mine	1-9	80	18.6
	10-24	282	65.6
	25+	68	15.8
How many trips back to Mozambique	1 – 3	220	51.3
	4 – 6	163	38.0
	7 – 9	17	4.0
	>10	29	6.8

^a^Excludes missing values for each factor

**Table 2:** Proximate, biological determinants and health outcomes^a^ among miners

		Number (430)	Percentage
*Proximate determinants*			
Age at first sex (recode)	< 18	194	45.1
	19-24	194	45.1
	≥ 25	42	9.8
Total number of sexual partners(recode)	One	208	48.7
	2	142	33.3
	3+	77	18.0
Condom use	Never	290	68.9
	Always/Sometimes	131	31.1
Paid for sex last 12	No	407	96.2
	Yes	16	3.8
Use of alcohol and drugs before sex_	Never	343	81.5
	Sometimes/Always	78	18.5
Ever circumcised	0	143	33.3
	1	287	66.7
*Biological determinants*			
Discharge in penis	No	413	96.3
	Yes	16	3.7
Sore or ulcer in penis	No	410	95.3
	Yes	20	4.7
Diagnosed STI	No	411	95.6
	Yes	19	4.4
Diagnosed with TB	No	406	94.4
	Yes	24	5.6
*Health outcome*			
Result of ELISA	0	247	77.7
	1	71	22.3

^a^Excludes missing values for each factor

### Factors associated with risky sexual behaviours among Mozambican miners

#### Multiple sexual partners

The odds of reporting one sexual partner were roughly three times higher for miners working as perforators as opposed to other types of occupation. Participation in an HIV lecture was significantly related with how many sexual partners were reported. The odds of having just one sexual partner for the miners participating in HIV lectures were roughly half (O.R. = 0.46) that of those who did not participate in HIV lectures.

#### Condom use

Younger miners were more likely to use condoms than older ones. In fact, the odds of condom use – always or sometimes – by miners in the 31-40 age group were three times higher than the odds of condom use in the 51+ age group. This again was controlled for other underlying factors.

Miners with lower education levels were less likely to use condoms. The odds of condom use – always or sometimes – for miners with primary education were approximately half (O.R. = 0.485) that of those with secondary or higher education. Moreover, condom use was more likely amongst known HIV+ miners. Indeed, the odds of condom use for known HIV- miners were 0.369 times greater than that of known HIV+ miners.

#### Paid sex

Controlling for other extenuating factors, living alone while working in South Africa increases the likelihood of engaging in paid sex. For miners living in hostels, the odds of paying for sex were lower by a factor of 0.207 compared to the odds when living alone.

#### Use of alcohol and drugs before sex

Married miners were less likely to use alcohol or drugs before sex compared to divorced or widowed men (O.R. = 0.039). For miners working as perforators, the odds of using alcohol or drugs before sex were approximately five times that of miners with other occupations, keeping the other underlying factors constant.

Miners who perceived their risk of HIV infection as low or medium were three to four times respectively more likely to use alcohol or drugs before sex compared with miners with known HIV+ status, given other underlying factors. Moreover, when controlling for the other underlying factors, living in a hostel or with a girlfriend/friends/colleagues, increases the odds for alcohol or drug consumption before sexual engagement, by three to five times respectively compared to living alone (Table 3).

**Table 3:** Odds ratio estimates for the association between proximate and underlying determinants among miners

	Total number of sexual partners within the previous yea		Condom use in last 3 sexual acts within the previous year		Had paid sex within the previous years		Age at first sex		Use of alcohol and drugs before sex within the previous year		Circumcision	
	Estimate	*p*-value	Estimate	*p*-value	Estimate	*p*-value	Estimate	*p*-value	Estimate	*p*-value	Estimate	*p*-value
Age Group (years)												
21 –30	1.917	0.248	0.566	0.427	0.000	0.998	**0.075**	**0.001**	0.247	0.133	1.569	0.548
31– 40	1.547	0.186	**3.086**	**0.004**	1.118	0.920	**0.282**	**0.000**	0.999	0.998	0.835	0.678
41 – 50	0.955	0.884	1.113	0.779	2.443	0.366	1.146	0.658	0.604	0.258	1.022	0.957
> 51 (reference)												
Marital status												
Never married	0.331	0.434	0.000	0.999	0.000	0.999	4.874	0.336	0.019	0.063	1.667	0.779
Married/Union	0.647	0.529	0.531	0.253	0.098	0.066	**9.469**	**0.019**	**0.039**	**0.000**	0.579	0.459
Divorced/Widow (reference)												
Marital status in SA and Moz												
Not married	0.000	0.999	1.968	0.375	1.957	0.622	0.000	0.999	3.516	0.059	1.197	0.778
One in SA	0.717	0.741	1.748	0.620	0.329	0.171	0.343	0.297	3.234	0.252	1.370	0.765
One in Moz	**0.236**	**0.000**	0.790	0.597	0.121	0.120	**0.318**	**0.004**	0.782	0.596	1.516	0.275
More than one in Moz	1.763	0.246	0.423	0.176	0.000	0.998	0.437	0.101	1.142	0.811	0.741	0.515
One in SA and Moz (reference)		.										
Province of residence in Mozambique												
Maputo	0.933	0.936	0.315	0.207	0.047	0.072	0.962	0.962	0.142	0.059	1.046	0.957
Gaza	0.969	0.970	0.458	0.378	0.053	0.082	1.425	0.657	0.248	0.157	0.833	0.826
Inhambane	1.332	0.734	0.262	0.134	0.067	0.096	0.728	0.691	0.171	0.073	**37.448**	**0.000**
Other (reference)												
Education level _ref.												
No Schooling	0.297	0.077	0.720	0.622	0.000	0.998	**3.367**	**0.043**	0.000	0.998	0.574	0.472
Primary	0.992	0.978	**0.485**	**0.034**	2.044	0.483	0.924	0.804	0.523	0.107	0.733	0.480
Secondary (reference)												
Occupation												
Engine driver	1.459	0.359	2.326	0.097	3.276	0.448	2.111	0.064	2.518	0.239	**0.310**	**0.024**
Perforator	**2.545**	**0.035**	1.694	0.332	2.033	0.672	1.224	0.650	**4.999**	**0.048**	**0.252**	**0.014**
Team leader	1.749	0.229	2.047	0.210	0.000	0.997	3.232	0.011	2.865	0.212	**0.265**	**0.022**
Outro (reference)												
Tested for HIV previously												
No	0.000	0.999	0.542	0.109	0.000	0.998	1.398	0.254	0.774	0.538	0.687	0.233
Yes (reference)												
Result of last HIV test												
Negative	0.849	0.705	**0.369**	**0.039**	3.463	0.354	1.916	0.157	0.346	0.106	1.817	0.262
Positive (reference)												
Participated in an HIV lecture												
Yes	**0.460**	**0.005**	0.646	0.174	0.346	0.139	0.601	0.078	0.966	0.932	1.022	0.955
No (reference)												
Perceived risk of contracting HIV												
None	0.640	0.267	0.456	0.100	0.232	0.215	0.479	0.077	0.460	0.347	0.750	0.551
Low	0.758	0.378	1.050	0.893	0.000	0.996	0.929	0.817	**3.077**	**0.019**	0.668	0.314
Moderate	0.932	0.805	0.866	0.668	0.539	0.403	0.975	0.932	**4.036**	**0.002**	1.412	0.383
High	0.000	0.999	0.000	0.999	0.000	0.999	0.000	0.999	0.000	0.999	0.000	0.999
HIV+ (reference)												
Accommodation in SA												
Hostel	1.066	0.805	1.044	0.886	**0.207**	**0.042**	1.387	0.233	**2.876**	**0.010**	1.434	0.293
Family	0.652	0.189	0.714	0.379	0.495	0.446	1.267	0.475	2.373	0.086	1.238	0.608
Girlfriend/ friend /Colleague	0.770	0.509	0.709	0.476	0.000	0.997	1.952	0.101	**5.323**	**0.002**	0.438	0.150
Alone (reference)												
Years working at the mine												
1 – 9	0.970	0.948	0.909	0.857	0.565	0.648	1.522	0.373	1.385	0.652	0.423	0.151
10 – 24	0.649	0.221	0.637	0.286	0.277	0.232	1.189	0.616	1.993	0.207	1.017	0.969
25+ (reference)												
How many trips back to Mozambique												
1 – 3	1.124	0.788	0.800	0.645	0.217	0.153	0.865	0.750	2.672	0.245	0.448	0.111
4 – 6	2.040	0.103	1.104	0.837	0.141	0.076	0.824	0.672	4.654	0.068	0.859	0.759
7 – 9	1.379	0.625	1.219	0.789	1.769	0.646	0.736	0.658	3.807	0.204	0.789	0.780
> 10 (reference)												

Test of proportional odds for number of sexual partners (*p*-value = 0.649)

Test of proportional odds for age at first sex (*p*-value = 0.274)

Significant estimates, at 5% significance level, are shown in "bold"

### Predictors of HIV infection among Mozambican miners

Model B reveals the odds of HIV positive status for those sometimes or always using condoms was 2.16 (95% C.I.: 1.13 - 4.12) times that of those never using condoms, controlling for the biological and other proximate determinants. The odds of being HIV positive for those reporting using alcohol or drugs before sex were 0.50 (95% C.I.: 0.22 - 1.15) times that of those not using alcohol or drugs before sex and 0.59 (95% C.I.: 0.32, 1.09) times for circumcised versus uncircumcised miners.

In Model C. odds ratios were estimated with the addition of underlying determinants of Model B. This included underlying determinants found to be significantly associated with the proximate determinants. After adjusting for proximate and underlying determinants, no significant association between HIV status and biological factors were found. However, the odds of being HIV positive for miners having 2 or more sexual partners is 0.29 times lower than the odds for miners reporting just one sexual partner in the last 12 months, allowing for other factors within the model.

Use of alcohol or drugs before sex also seems to reduce the likelihood of HIV infection. The odds of being HIV positive when the miner reports use of alcohol or drugs – sometimes/always – is 0.32 times lower than the odds for those who never use alcohol or drugs, when controlling for biological, proximate and underlying determinants (Tables 4 and 5).

**Table 4:** Bivariate analysis of HIV status and biological, proximate and underlying determinants among miners

		Result of ELISA				
		Negative		Positive		
		Number	Percentage	Number	Percentage	O.R. (95% C. I.)
Discharge in penis	No	239	78.5	65	21.5	1.00
	Yes	11	83.5	2	16.5	0.720 (0.164 – 3.163)
Sore or ulcer in penis	No	236	78.6	64	21.4	1.00
	Yes	14	75.8	5	24.2	1.175 (0.393 – 2.383)
Diagnosed with STI	No	236	78.1	66	21.9	1.00
	Yes	14	85.8	2	14.2	0.590 (0.146 – 2.383)
Diagnosed with TB	No	242	80.0	61	20.0	1.00
	Yes	8	49.3	8	50.7	**4.118 (1.474 – 11.507)**
Total number of sexual partners in 12 months	1	112	79.1	29	20.9	1.00
	2	86	77.0	26	23.0	1.131 (0.621 – 2.060)
	3+	52	79.2	14	20.8	0.993 (0.482 – 2.046)
Condom use in last 3 sexual acts	Never	178	81.2	41	18.8	1.00
	Always/Sometimes	68	71.4	27	28.6	1.729 (0.989 – 3.020)
Paid for sex last 12 months	No	235	78.9	63	21.1	1.00
	Yes	11	67.0	5	33.0	1.849 (0.625 – 5.471)
Use of alcohol and drugs before sex	Never	200	77.1	59	22.9	1.00
	Sometimes/Always	47	83.4	9	16.6	0.672 (0.314 – 1.437)
Age at first sex	≤18	117	77.8	33	22.2	1.00
	19-24	106	78.0	30	22.0	0.989 (0.566 – 1.728)
	≥25	27	84.0	5	16.0	0.668 (0.240 – 1.856)
Circumcision	No	68	71.8	27	28.2	1.00
	Yes	182	81.3	42	18.7	0.585 (0.334 – 1.023)
Age	> 51	77	79.7	20	20.3	1.00
	21 – 30	19	81.6	4	18.4	0.882 (0276 – 2.823)
	31 – 40	77	74.1	27	25.9	1.367 (0.706 – 2.649)
	41 – 50	77	81.3	18	18.7	0.903 (0.442 – 1.847)
Marital status in SA and Moz	1 in SA and Moz	20	80.7	5	19.3	1.00
	No wife	10	68.0	4	32.0	1.962 (0.439 – 8.766)
	1 in SA	4	78.6	1	21.4	1.137 (0.101 – 12.77)
	1 in Moz	193	79.7	49	20.3	1.066 (0.377 – 3.011)
	> 1 in Moz	24	72.4	9	27.6	1.593 (0.455 – 5.575)
Education level	Secondary	34	80.7	8	19.3	1.00
	None	13	81.9	3	18.1	0.926 (0.206 – 4.172)
	Primary	203	77.9	58	22.1	1.184 (0.523 – 2.677)
Occupation	Other	24	82.9	5	17.1	1.00
	Engine driver	132	77.2	39	22.8	1.429 (0.508 – 4.018)
	Perforator	56	82.2	12	17.8	1.052 (0.333 – 3.324)
	Supervisor	38	75.2	12	24.8	1.596 (0.499 – 5.108)
Perceived risk of contracting HIV	HIV+	1	4.9	19	95.1	1.00
	None	28	84.4	5	15.6	**0.009 (0.001 – 0.091)**
	Low	58	82.0	13	18.0	**0.011 (0.001 – 0.096)**
	Moderate	79	84.3	15	15.7	**0.010 (0.001 – 0.081)**
	High	73	82.4	16	17.6	**0.011 (0.001 – 0.092)**
Accommodation in S.A.	Alone	79	81.0	18	19.0	1.00
	Hostel	103	79.0	27	21.0	1.136 (0588 – 2.195)
	Family	45	74.5	15	25.5	1.459 (0.677 – 3.144)
	Girlfriend/friends	23	76.4	7	23.6	1.318 (0.496 – 3.504)

Significant estimates, at 5% significance level, are shown in "bold"

**Table 5:** Odds ratio (95% C.I) of HIV status for proximate and biological determinants

	Models A (*n* = 317)			Model B (*n* = 310)			Model C (*n* = 297)		
Factors (reference category)	O.R.	95% C.I.		O.R.	95% C.I.		O.R.	95% C.I.	
Discharge in the penis (No)									
Yes	0.53	0.09	2.86	0.55	0.09	3.28	0.69	0.06	7.57
Sore or ulcer in the penis (No)									
Yes	2.46	0.59	10.19	2.87	0.61	13.61	2.14	0.32	14.11
Diagnosed with STI (No)									
Yes	0.38	0.07	1.96	0.28	0.05	1.67	0.23	0.02	2.18
Diagnosed with TB (No)									
Yes	**4.55**	1.60	12.93	**3.88**	**1.23**	**12.5**	1.84	0.28	12.15
Total number of sexual partners in 12 months [1]									
2				0.89	0.46	1.76	0.57	0.23	1.44
3+				0.58	0.24	1.37	**0.29**	0.09	0.98
Condom use in last 3 sexual acts (Never)									
Sometimes/Always				**2.16**	1.13	4.12	2.02	0.87	4.66
Paid for sex last 12 months (No)									
Yes				1.65	0.49	5.53	3.57	0.83	15.43
Use of alcohol and drugs before sex (Never)									
Sometimes/Always				0.50	0.22	1.15	**0.32**	0.11	0.98
Age at first sex (≤ 18)									
19 – 24				0.85	0.47	1.55	0.82	0.38	1.78
≥ 25				0.54	0.18	1.60	0.79	0.22	2.79
Circumcision (No)									
Yes				0.59	0.32	1.09	0.59	0.28	1.22
Age (≥ 51)									
21 – 30							1.17	0.24	5.82
31 – 40							1.49	0.62	3.62
41 – 50							0.60	0.23	1.61
Number of wives in both countries (1 in SA and Moz)									
No wife							2.05	0.27	15.61
1 in SA							0.33	0.01	13.71
1 in Moz							0.89	0.22	3.65
> 1 in Moz							2.05	0.41	10.21
Education level (Secondary+)									
None							0.45	0.05	3.78
Primary							0.74	0.26	2.09
Occupation (other)									
Engine driver							2.42	0.52	11.28
Perforator							2.89	0.56	15.01
Supervisor							3.61	0.68	19.18
Perceived risk of contracting HIV (HIV+)									
None							**0.004**	0.000	0.05
Low							**0.005**	0.000	0.06
Moderate							**0.007**	0.001	0.08
High							**0.005**	0.000	0.06
Accommodation in S.A. (alone)									
Hostel							1.58	0.66	3.76
Family							1.64	0.56	4.81
Girlfriend/friends							1.62	0.462	5.67

Significant estimates, at 5% significance level, are shown in "bold"

## Discussion

Our findings suggested there is a great need to shift the actual thinking about how to change sexual behaviour. The current position is change will occur as a result of personal/individual decision making. However, the complexity of factors possibly influencing sexual behaviours under the socio-economic and contextual circumstances in which miners are inserted, stresses the need for HIV prevention strategies to transcend a unique focus on individual factors. Therefore broader social and contextual phenomena influencing HIV risk in Mozambican miners must be considered.

For instance, the literature demonstrates male collective arrangements, such as hostels, are fertile environments for men to engage in alcohol and/or drug use as well as risky sexual behaviour [21]. Our study indicates living in a hostel and with girlfriend/friends or colleagues increases the likelihood for alcohol or drug consumption before sex engagement, by three to five times respectively compared to living alone. Nevertheless, miners who live alone, in South Africa were more likely to engage in paid sex. Poudel’s study [22] reported migrants often seek sexual partners to relieve their loneliness and to distract themselves from anxieties about home.

Furthermore, the type of occupation at the mining operation seems also to influence miners’ risky sexual behaviour. For instance, the odds of using alcohol or drugs before sex for miners working as perforators were approximately five times that of miners with other occupations. The odds of reporting one sexual partner were roughly three times higher for miners working as perforators as opposed to other types of occupations.

Campbell [23] suggested working conditions in South African mines are harsh, and often lead to extremely high stress situations. Therefore, workers need to unwind and relax at the end of the day, so drinking and sex are the most available diversionary activities. Additionally, the close association between alcohol use and unsafe sexual practices is well documented.

In HIV interventions, both in Mozambique and South Africa, the role of social and structural environment has been neglected. Interventions attempt to change an individual’s motivations without addressing the root causes or the context which encourages HIV risk-taking among miners. Thus, HIV interventions seem to be effective in increasing risk perception regarding HIV and often reducing miners’ propensity for risky sexual behaviour among those who are already infected by HIV.

However, it seems to not be effective enough to change HIV-related risk behaviours among those who are not yet infected. For instance, miners who perceived their risk of HIV infection as low or medium were three to four times more likely to use alcohol or drugs before sex than miners with known HIV+ status.

Apart from this, it seems HIV counselling and the knowledge shared in the art consultations cause HIV positive people to engage in more safe sex behaviour. On one hand, our results revealed the odds of engaging with multiple sexual partners or alcohol-related risk is lower for positive miners than for their negative counterparts. On the other hand, the odds of using condoms are higher for HIV positive miners compared to negative miners. These results reinforce our views stated in a previous discussion – knowing their HIV positive status moves miners toward safer sex behaviour.

Our findings also revealed socio-demographic factors, such as age, marital status and educational level are predictors of risky sexual behaviour among Mozambican miners. Therefore, we argue the nature of existing circular migration from Mozambique to South Africa requires an integrated approach of on-going interventions at both ends of the migration spectrum. It is essential migrants and migration are not exceptionalised when considering HIV programming. Migrants and non-migrants form overlapping sexual networks and require linked responses [24].

### Study limitations

Many research issues must be addressed before we can better understand HIV transmission in certain populations in the context of generalised epidemics such as is the case in Mozambique. Little is known about how to best measure underlying variables at the individual or group levels, or how to best take into account the effects of interactions among such variables.

In this instance, interactions are likely to be critical. Similar issues regarding measurement in the proximate determinants and their influence on biological determinants are even more difficult to quantify. This is because the proximate and biological determinants are self-reported sexual behaviours. They cannot be measured directly, only be inferred. Understandably, this may give rise to concerns about recall bias and social desirability tendencies. Furthermore, these datasets are the result of cross-sectional behavioural surveys, which prohibits inference of causality.

Apart from this, the majority of mineworkers included in this study were from the southern region of Mozambique. Therefore, our results were unable to analyse geographic differences throughout the country. Lastly, the study sample size estimate was based on the surveillance goal of detecting major changes in the epidemic over time, not in the number of Mozambican miners working in South Africa. Therefore it may have lacked representativeness of the population of the active Mozambican miners working in South Africa, thus reducing the generalisability of our results.

Apart from that, active miners were selected, in Ressano Garcia Border. This is where these migrant labourers travelled to renew or sign new contracts enabling them to work in South African mines.

Former miners and other fractions of the miner community - such as those who have abandoned mining work for different reasons, those who are currently not working but living in their place of residence, including those that suffer from HIV/AIDS or other HIV-related diseases and could not return to the mines - were not included in the study. Perhaps these particular people were the healthiest of all mine workers. Therefore, we were not able to provide a complete picture of the miner community or the impact of HIV/AIDS among them, resulting in “the healthy worker survivor” effect.

## Conclusions and implications

In Mozambique, HIV responses have been dominated by biomedical/behavioural strategies, prompting biomedical/behavioural solutions. These strategies do not take into adequate account the social factors shaping the biomedical and behavioural dimensions of HIV/AIDS amongst miners. Therefore the likely result is an unduly limited range of responses to the problem. Therefore, rather than focus only on a behavioural science approach, we suggest broadening the scope to include comprehensive HIV intervention approaches, including structural, environmental, and individual levels.

We recognise mass awareness programs concerning condom use for HIV prevention is especially needed for those who are not aware of their HIV status as well as those who are aware they are not positive. Such sessions would include interventions and provide individual or group counselling to the miners on the topic of HIV risk associated with unprotected sex with female sex workers. Additionally, increased provision of HIV testing for miners would be a viable programmatic strategy for attaining significant increases in condom use.

However, structural interventions are also feasible and effective. This is especially so when they are bolstered by political, organisational and governmental policies [8]. In this specific case, more family-friendly contracts would allow migrant miners more engagement with their families. Therefore, the social tissue would be less likely to be broken by their long absences from home. Employers should also be encouraged to improve working and living conditions at the workplace.

Furthermore, multilevel alcohol Venue-based HIV interventions – that is, interventions targeted at individuals and their settings [25] – may be required to help prevent HIV transmission among miners.

Moreover, interventions targeting both the general population and specific sub-populations, including miners and their families, should be combined. Increased access to health facilities for miners and the general population of Mozambique is also urgently needed. Such interventions would primarily target miners who are married, less educated and those who have wives in both Mozambique and South Africa.

Additionally, there may be other factors needing to be identified and addressed first. Thus, future research is highly recommended to gain a better understanding of miners’ HIV risks. A second round of IBBS among miners is planned for later in 2017. Thus, the results can be compared to the first round to provide a more comprehensive risk profile of miners and HIV risk-behaviours.

## Abbreviations

AIDS: Acquired immunodeficiency syndrome
HIV: Human immunodeficiency virus
IBBS: Integrated biological and behavioural survey
STIs: Sexually transmitted infections
TB: Tuberculosis
TEBA: The employment Bureau of Africa
TLS: Time location sampling
